# Syntenin-knock out reduces exosome turnover and viral transduction

**DOI:** 10.1038/s41598-021-81697-4

**Published:** 2021-02-18

**Authors:** Rudra Kashyap, Marielle Balzano, Benoit Lechat, Kathleen Lambaerts, Antonio Luis Egea-Jimenez, Frédérique Lembo, Joanna Fares, Sofie Meeussen, Sebastian Kügler, Anton Roebroek, Guido David, Pascale Zimmermann

**Affiliations:** 1 Department of Human Genetics, KU Leuven, Leuven, Belgium; 2 Aix-Marseille Univ, Inserm, CNRS, Institut Paoli-Calmettes, CRCM, Equipe Labellisée LIGUE 2018, Marseille, France; 3 Department of Neurology, University Medicine Göttingen, Göttingen, Germany

**Keywords:** Extracellular signalling molecules, Glycobiology

## Abstract

Exosomal transfers represent an important mode of intercellular communication. Syntenin is a small scaffold protein that, when binding ALIX, can direct endocytosed syndecans and syndecan cargo to budding endosomal membranes, supporting the formation of intraluminal vesicles that compose the source of a major class of exosomes. Syntenin, however, can also support the recycling of these same components to the cell surface. Here, by studying mice and cells with syntenin-knock out, we identify syntenin as part of dedicated machinery that integrates both the production and the uptake of secreted vesicles, supporting viral/exosomal exchanges. This study significantly extends the emerging role of heparan sulfate proteoglycans and syntenin as key components for macromolecular cargo internalization into cells.

## Introduction

Exosomes are small vesicles of endosomal origin that can transfer cellular contents (i.e. mRNA, miRNA, lipids, receptor proteins, small GTPases, kinases) between cells^1–3^. Currently, exosomal transfers are recognized as an important means of intercellular communication, modulating various physiological and pathophysiological processes^4–8^. In particular, vesicular transfers between cells are implicated in brain disease and mechanisms of neurodegeneration^9–12^. Specifically, extracellular vesicles isolated from the brains of transgenic mice that express human four repeat tau with the P301L mutation that is linked to hereditary tauopathy seed tau protein aggregation^13^. Exosomes isolated from the brains of these animals are taken up by neurons and hijack the endosomal pathway to spread to interconnected neurons^14^. Finally, neutral sphingomyelinase-2 inhibitor, preventing exosome production, halts tau propagation in the brains of mice injected with adeno-associated virus (AAV) expressing the mutant tau transgene in a neuron-specific manner^15^. Of note, several aggregation-prone proteins, such as beta-amyloid, tau, alpha-synuclein and prion protein are—at least in part—released from cells via unconventional secretion, i.e. extracellular vesicles/exosomes^16,17^. Thus, exosomal transfers might have wide implications and be of larger significance.

Exosomal transfers depend on both the biogenesis of exosomes in donor cells and the uptake of exosomes and retrieval of their contents by recipient cells. Globally, and even in part mechanistically, exosomal exchanges are reminiscent of viral infection^18,19^. Yet, exosomes are clearly heterogeneous in composition and properties^20–22^, and in the mechanisms of their biogenesis^6^. We identified syntenin as a platform for the formation of a major class of exosomes^23,24^. Syntenin is a small scaffold protein that, via its two PDZ domains, binds avidly to the cytosolic domains of clustered syndecans^25–27^. Syndecans are abundant and versatile co-receptors, their heparan sulfate (HS) moieties binding a multitude of components, with pleiotropic effects^28,29^. Via LYPX_n_L sequences, as occur also in some viral late domains, syntenin binds also to ALIX and associated ESCRT proteins^23^, membrane-bending and abscission machinery that supports the formation of endosomal intraluminal vesicles^30^ and is exploited by viruses for budding and escape from cells^31,32^. In a process that requires ARF6 and the ARF6-effector PLD2^33^ and by adapting syndecans to ALIX and ESCRTs, syntenin directs endocytosed syndecans and syndecan-bound cargo to budding endosomal membranes, supporting the formation of intraluminal vesicles (ILV) and exosomes^23^. Heparanase, trimming the HS-chains on syndecan in the lumen of the endosomes^34^, and the cytosolic tyrosine-phosphorylations of both the syndecan-intracellular domain (ICD) and syntenin by c-src^35^ markedly stimulate the formation of syntenin-exosomes. In effect, loss-of-syntenin markedly compromises ILV formation and exosome production^23^.

However, in a process that also depends on ARF6, but requires PIPK5 as ARF6-effector and syntenin to bind also PIP2, syntenin also supports the recycling of syndecans and syndecan cargo back to the cell surface, avoiding their exosomal/lysosomal destinations and potentially regulating their cell surface abundance^36^. At cell surfaces, syndecans interact with a large number of proteins, including growth factors, chemokines and structural proteins of the extracellular matrix and cognate receptors, to influence cell adhesion, growth and differentiation and cellular responses to the environment^28,29^. Of note, heparan sulfate proteoglycans (HSPGs) also function as internalizing receptors for various macromolecular cargo, including viruses and extracellular vesicles such as exosomes^37–40^. Internalized exosomes co-localize with vesicles containing HSPGs (including syndecans), and their capture and internalization depend on intact HSPG synthesis and HS-sulfation in recipient cells. Syntenin loss-of-function, therefore, potentially also affects the retrieval of information that might be embodied in exosomes.

Here, we investigate this contention through the construction of mice and cells with syntenin-knock out (KO). Mice with syntenin-KO show no overt abnormalities. However, when providing the genetic background for a model of tau-mediated neurodegeneration that is based on the AAV6-mediated expression of mutant P301L protein tau, compared to wild-type animals, mice with syntenin-KO were found to express recombinant human tau at much lower levels and in a more restricted area of the brain. To explore this further, we established MEFs from these animals. We found that, compared to wild-type MEFs, syntenin-KO MEFs are less easily transduced by recombinant retrovirus. MEFs with syntenin-KO also internalize significantly lower amounts of cancer cell-derived exosomes. MCF-7 cells with CRISPR/Cas9-mediated syntenin-KO (Synt-CRISPR) mimic these defects. Consistent with the role of syntenin in SDC recycling, MEFs and MCF-7 cells with syntenin-KO show markedly reduced expressions of syndecan core proteins and cell surface HS. Yet, syntenin-KO also results in a decrease of syndecan mRNAs. Increasing the levels of syndecan and HS in cells increases the susceptibility of the cells to retroviral transduction. Yet, like wild-type syntenin, a recycling-defective syntenin rescues retroviral transduction, while a syntenin defective for ALIX-binding and endosomal budding/exosomal secretion fails to do so. Taken together, our data reveal that syntenin not solely controls the production of exosomes, but is also required for the retrieval of information embodied in viruses/exosomes. This study significantly extends the emerging role of syntenin and HSPGs as key components for the exchange of macromolecular cargo between cells.

## Results

### Syntenin knock-out dampens AAV-mediated tau expression in the brain of mice

**Figure 1:**
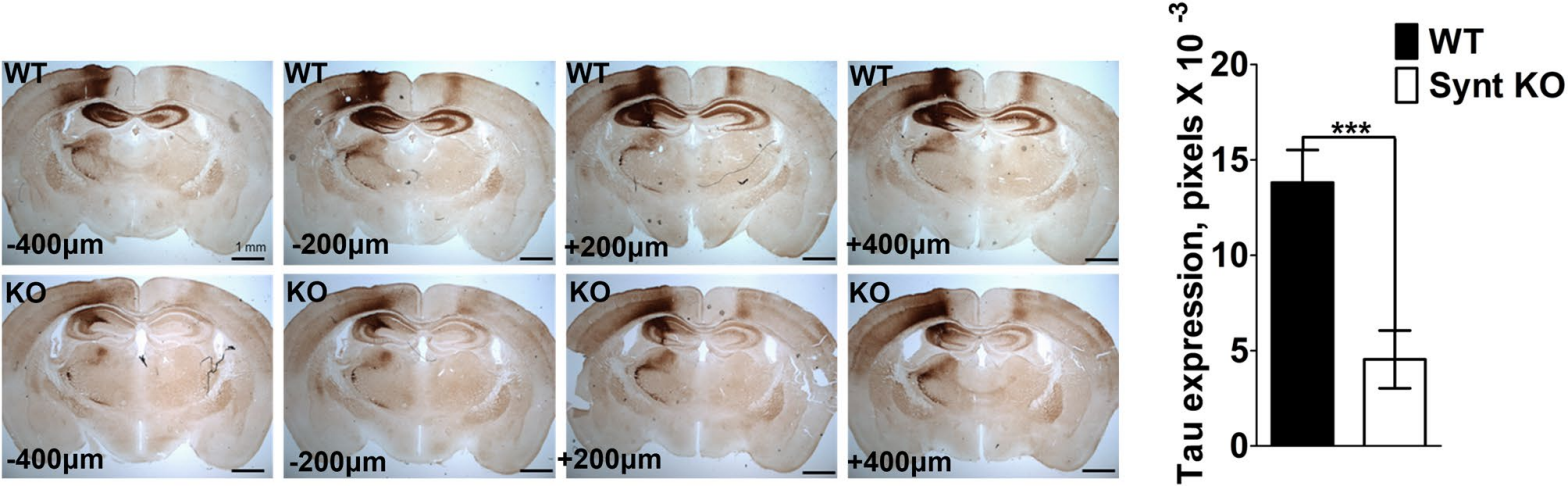
Syntenin-knock out reduces the AAV-mediated expression of tau-P301L in mouse brain. Micrographs of brain sections illustrating the expression of human tau protein after intracerebral injection of AAV-6-tau4R-P301L viral particles in the left hemisphere of wild-type mice (WT, top) or syntenin-knock out mice (KO, bottom). Note the low expression of tau in KO brain, both at caudal (−) and rostral (+) positions relative to the site of injection. Histogram on the right indicates the number of ‘dark’ pixels (with density above an arbitrarily set threshold) per complete brain section (left + right hemispheres). Data were collected using 4 mice per group and 4 sections per mice. Values are expressed as mean ± SEM. Scale bar is 1 mm.

To study the in vivo effects of a disruption of syntenin function, we developed a mouse strain with a constitutive general syntenin-KO. Such mice present no overt anomalies (Supplemental Fig. 1). As the development of tauopathy might depend on exosomes^8,15–17^, we were interested in testing whether the loss of syntenin affects the outcome of the intracerebral injection of adeno-associated viral (AAV) vector encoding tau-P301L. As expected, the IHC analysis of the brains of wild type animals revealed the expression of large amounts of human tau (Fig. 1, top panels). In comparison, the brains of syntenin-KO animals expressed much lower levels of human tau (Fig. 1, bottom panels). Such expression deficit occurred on both the rostral and caudal sides of the site of injection (Fig. 1, right versus left panels), and globally the tau signal was estimated at one third of that in wild type brain (Fig. 1, bar graph). These data suggest that syntenin KO animals might be ‘refractory’ to viral infection or to the cellular consequences of the viral transduction, or both.

### Primary mouse embryonic fibroblasts and MCF-7 cells with syntenin-KO

To start exploring a possible basis for the above findings in animals, we established primary cultures of fibroblasts derived from wild-type (WT) and syntenin-KO mouse embryos (WT/KO-MEFs). At least in vitro, in cultured cell lines, and MCF-7 cells in particular, syntenin supports the vesicular trafficking of syndecans and syndecan-assisted receptor cargo, including receptors for adhesion molecules and growth factors. Syntenin is involved in directing endocytosed syndecan and syndecan cargo to budding endosomal membranes, ILVs and exosomes^23^ potentially sustaining signaling *in trans*, but also recycles these from endosomes back to the cell surface^36^, potentially sustaining signaling *in cis*.

**Figure 2:**
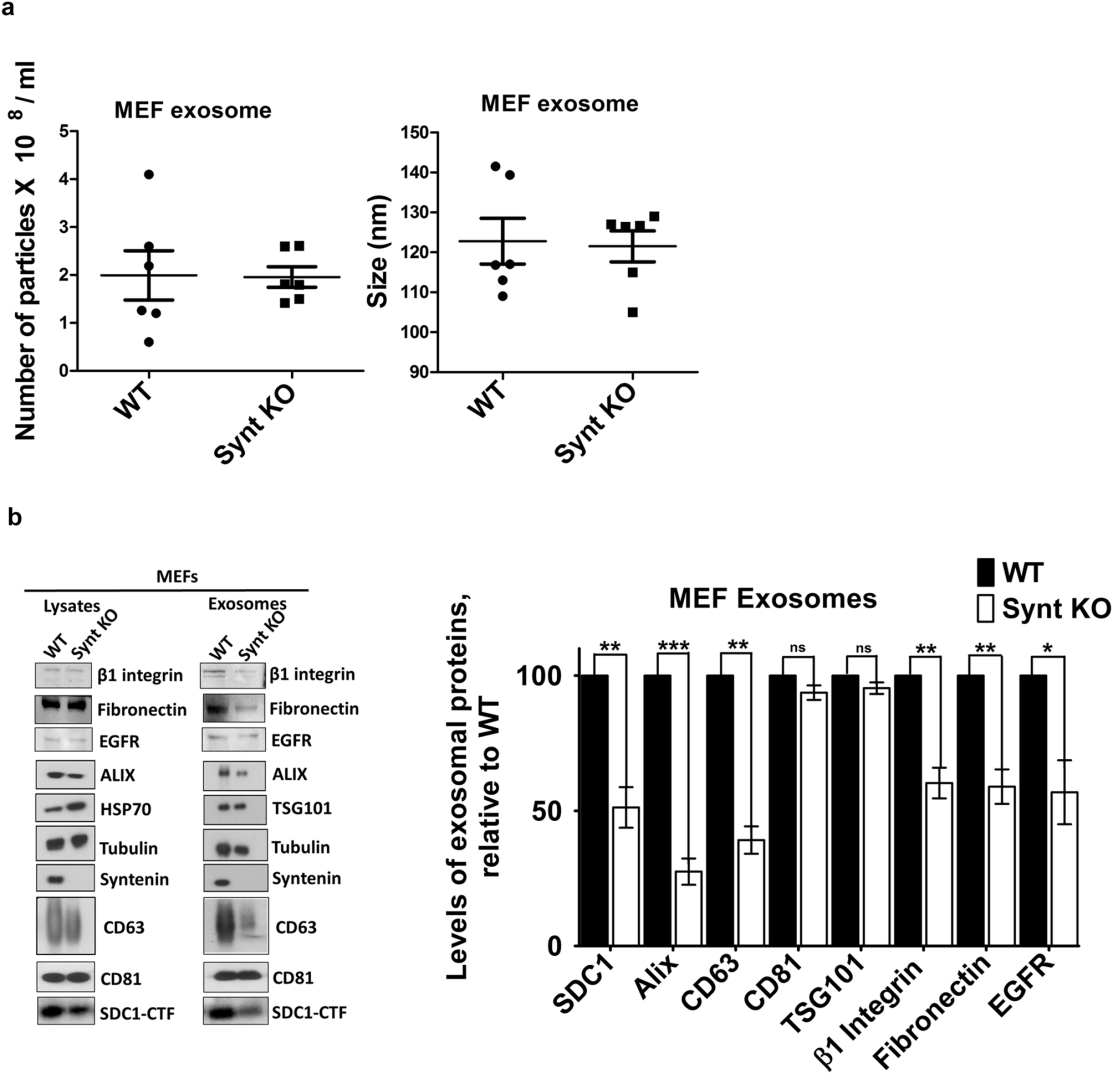
Syntenin-knock out reduces the exosomal secretions of syntenin cargo by primary MEFs in culture. (**a**) Exosome characterization by nanoparticle-tracking. Exosomes were collected from equivalent amounts of culture medium, conditioned by equal numbers of cells, for equal lengths of time. Particle numbers and sizes were analyzed by Nanosight. (**b**) Left: Western blot analysis of proteins present in the lysates and exosome preparations of WT and Synt KO MEFs in primary culture. Exosomes were collected from equivalent amounts of culture medium, conditioned by equal numbers of cells, for equal lengths of time. Right: Bar graphs showing the levels of exosomal marker proteins in Synt-KO MEFs, relative to the levels measured in WT cells (taken as 100%). Note the concurrent decreases of ALIX, CD63 and syndecan 1-CTF (SDC1-CTF), and potential syndecan cargo such as B1-integrin, fibronectin, and EGFR, but not CD81 and TSG101, in the exosomes of Synt KO MEFs.

**Figure 3:**
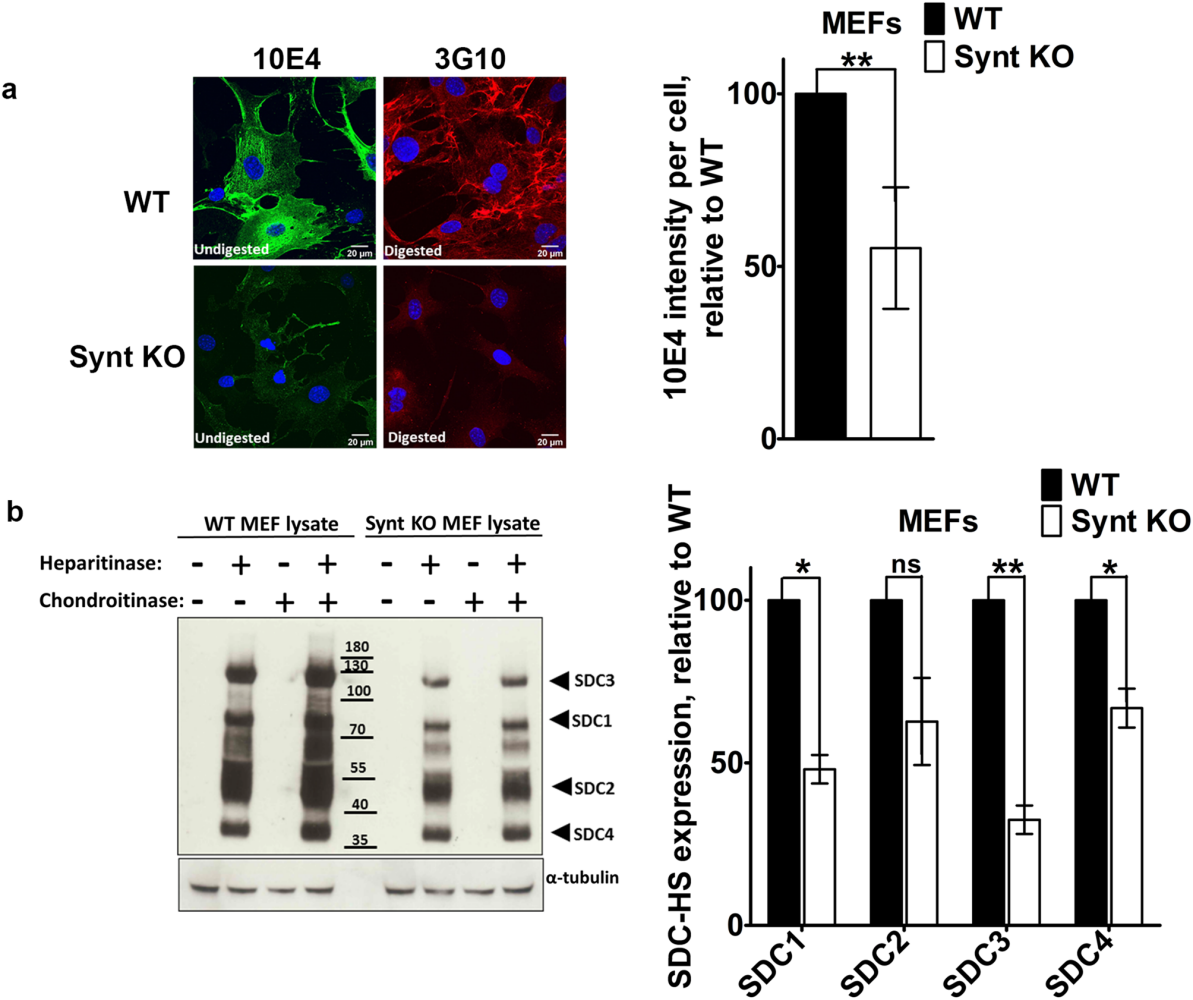
Loss of syntenin expression in primary MEFs leads to a decrease in HSPG expression. (**a**) Cell surface heparan sulfate abundance. Staining of total native HS, with mAb 10E4, in untreated cells (left panels), and of the residual delta-HS, with mAb 3G10, in cells treated with both heparitinase and chondroitinase ABC (right panels), in WT (top panels) and Synt-KO (bottom panels) MEFs. Compared to WT MEFs, Synt-KO MEFs show less intense fluorescence, for both 3G10 and 10E4, suggesting a reduction in both the number of HS chains and total mass of HS present at cell surfaces. Bar graphs on the right correspond to relative 10E4 fluorescence intensity per cell (taking WT cells as 100%). (**b**) Heparan sulfate proteoglycan abundance. (Left) Western blot of cell lysates, either left undigested (−) or digested (+) with heparitinase, chondroitinase ABC or a combination of both enzymes, and stained for delta-HS, using mAb 3G10, revealing the expression of HSPGs in WT and Synt-KO MEFs. Note that, compared to WT cells, Synt-KO MEFs show lesser amounts of delta-HS tagged PG core proteins (tentatively identified as the syndecans 1–4). (Right) Bar graphs represent the quantification of three independent experiments, taking band intensities in WT cells as 100%.

Nanosight analyses indicated that both types of MEF release similar numbers of particles, and that these particles are of similar mean sizes (Fig. 2a). Yet, compared to wild-type MEFs, MEFs with syntenin-KO produce exosomes that are less loaded with CD63 and SDC, cargo interacting directly with syntenin^26,41^, and less loaded with potential TSPN-SDC interacting cargo, such as integrins, fibronectin and EGFR (Fig. 2b). KO-MEFs also display lower levels of HS at their cell surfaces (Fig. 3a) and lower cellular levels of SDC (Fig. 3b, Supplemental Fig. 2). Noteworthy, MCF-7 cells with syntenin-KO, constructed by CRISPR/Cas9 technology (Synt-CRISPR MCF-7 cells) present similar defects (Supplemental Fig. 3). Thus, at least in the long term, the loss of syntenin seems to have pronounced effects on the cellular levels of direct syntenin cargo (i.e. SDC and CD63).

### Loss of syntenin expression limits retroviral transduction and exosome uptake

**Figure 4:**
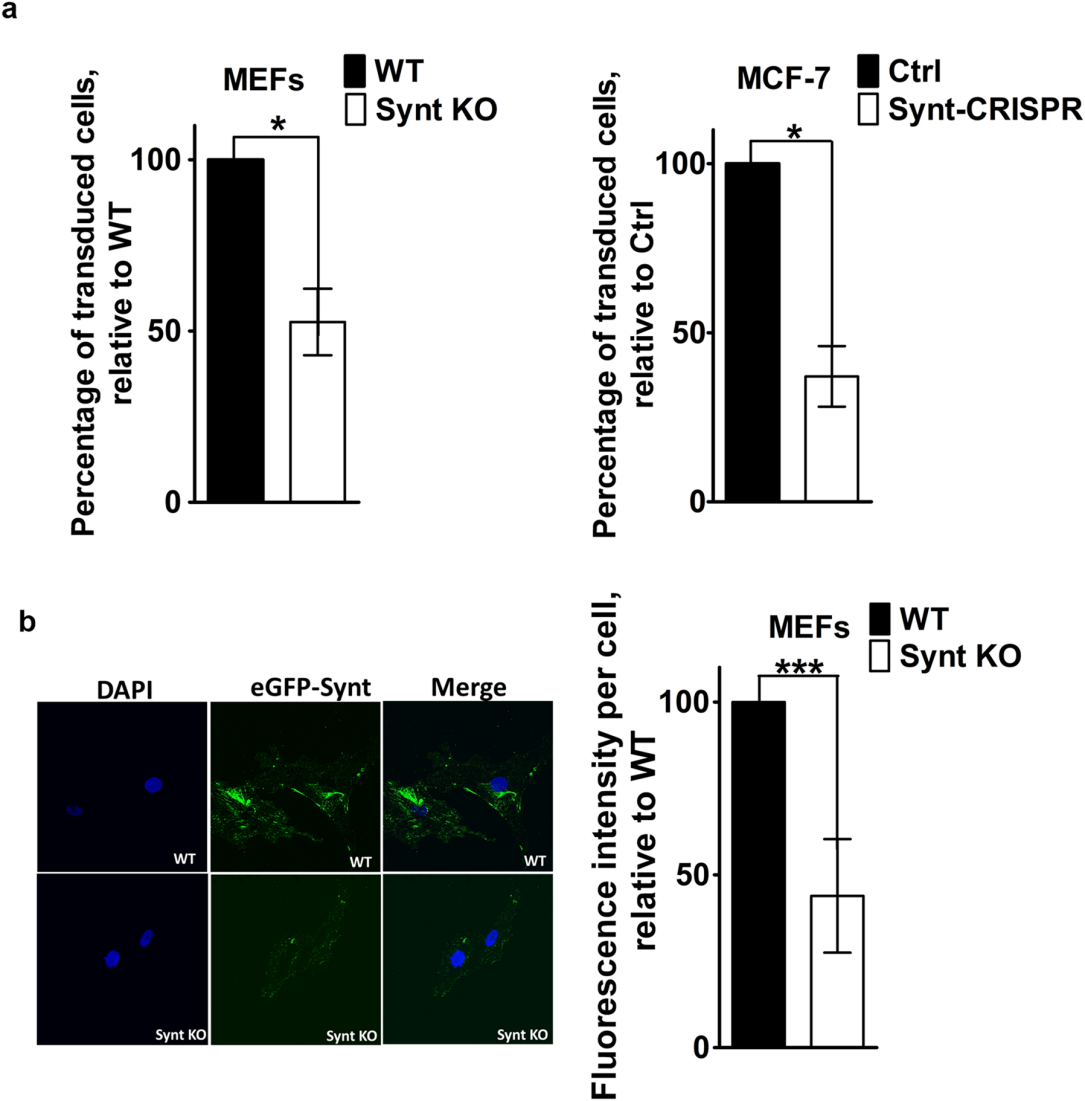
Loss of syntenin expression in primary MEFs limits retroviral transduction and exosome uptake. (**a**) Retroviral transduction. Retrovirus encoding LUC IRES eGFP, produced using phoenix packaging cells, was incubated with MEFs (WT and with Synt KO, shown on the left) and with MCF-7 cells (Ctrl and Synt-CRISPR, shown on the right), for 48 h. Cells expressing eGFP were quantified by flow cytometry. Bar graphs represent the percentage of cells transduced by retrovirus (i.e. expressing eGFP), relative to the percentage of WT cells that was transduced (taken as 100%). (**b**) Exosome uptake. MEFs, either WT or Synt-KO, were incubated with exosomes derived from MCF-7 cells expressing eGFP-syntenin (eGFP-Synt), at 37 °C for 8 h, to allow for exosome uptake, and then subjected to confocal laser scanning microscopy. Left: Representative confocal micrographs of MEFs, showing the accumulation of eGFP-Synt (green) and DAPI (blue) staining of the nuclei. Exosomes loaded with eGFP-Synt yield more puncta in WT cells than in Synt-KO cells. Right: quantification of mean eGFP fluorescence per cell.

We surmised that AAV-vector-mediated in vivo expressions of human tau might be lower in syntenin-KO than in control animals because viral transduction is less efficient in the absence of syntenin. To test this possibility, MEFs were infected with LUC IRES eGFP retrovirus and, after 48 h, the fraction of the cells that was (transduced and) expressing eGFP was measured. Syntenin-KO reduced eGFP-expression by ~ 48%, taking the percentage of GFP-expressing cells in WT cultures as 100% (Fig. 4a, left). Similar results were obtained in MCF-7 cells, observing a ~ 72% decrease in the percentage of eGFP-positive cells in Synt-CRISPR MCF-7 cell cultures (Fig. 4a, right). Since viral transduction represents a paradigm for exosome-mediated transfers, we also compared the MEFs for ‘cancer cell’ exosome uptake. For that, we incubated the MEFs with eGFP-labeled exosomes, derived from MCF-7 breast cancer cells that express a doxycycline-inducible eGFP-syntenin fusion protein (Supplemental Fig. 4), and quantified the amount of fluorescence (eGFP) inside (i.e. internalized by) the cells, using confocal microscopy (Fig. 4b). The mean level of eGFP per cell was approximately 64% lower in syntenin-KO MEFs, compared to WT MEFs (Fig. 4b, right). Taken together, these data indicate that syntenin supports viral transduction and exosomal uptake in recipient cells.

### ALIX-interacting syntenin rescues retroviral transduction in syntenin-negative MCF-7 cells

**Figure 5:**
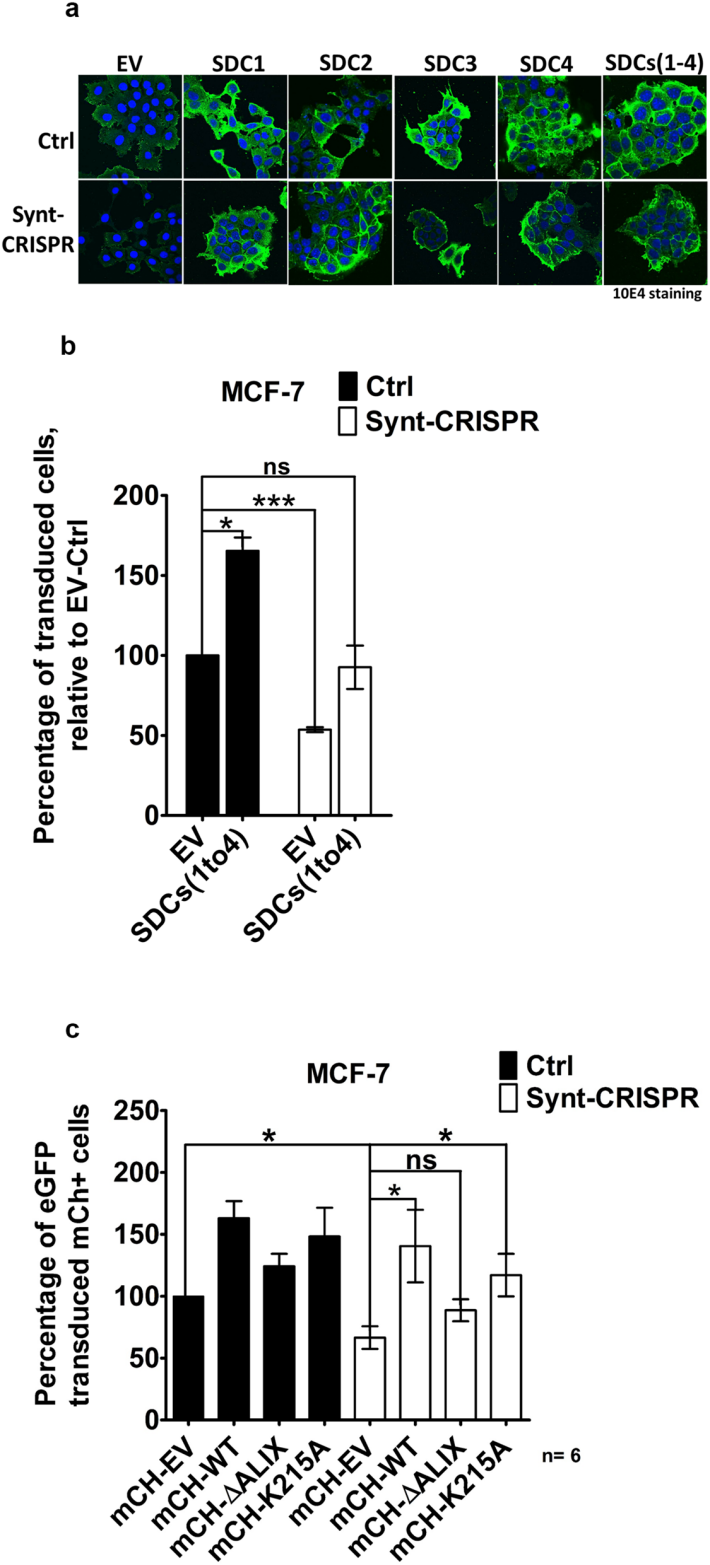
ALIX-binding syntenin rescues retroviral transduction in syntenin 1-negative MCF-7 cells. (**a**) Representative confocal micrographs of MCF-7 cells showing the distribution of heparan sulfate (as detected by mAb 10E4, green) and the DAPI (blue) staining of the nuclei, upon syndecan over expression (syndecans 1–4, individually and all four together in co-transfection) in control (Ctrl) and in syntenin 1-negative (Synt-CRISPR) MCF-7 cells. (**b**) Retroviral transfection was analyzed using flow cytometry. Retrovirus encoding LUC IRES eGFP, produced using phoenix packaging cells, was incubated for 48 h with Ctrl and with Synt-CRISPR MCF-7 cells, all or not over-expressing syndecans. Cells expressing eGFP were quantified by flow cytometry. (**c**) Wild-type MCF-7 cells and Synt-CRISPR MCF-7 cells were transfected with expression plasmid vector encoding mCherry (empty vector), mCherry-syntenin (wild-type syntenin), mCherry-syntenin ∆ALIX (syntenin defective in ALIX-binding) or mCherry-syntenin K215A (syntenin defective in cargo recycling), replated and then incubated for 48 h with retrovirus encoding LUC IRES eGFP. Fluorescent protein expressions were quantified by flow cytometry. eGFP expression in mCherry-expressing cells was taken as a measure of syntenin effects on retroviral transduction. In every experiment, the percentage of wild type cells transfected with empty vector and expressing mCherry that were expressing eGFP was taken as 100 percent. *n* = 6, bars represent mean values ± SD; n.s., non-significant, *P < 0.05, **P < 0.01, ***P < 0.001 (Student’s *t* test).

Similar to what we observed with MEFs (Fig. 3a), the KO of syntenin also reduces the HS expressions of MCF-7 cells (Fig. 5a, Supplemental Fig. 3b). To explore whether syndecans support HSPG-dependent retrovirus infection, retroviral transduction was analyzed in control and in Synt-CRISPR MCF-7 cells, when over-expressing syndecans (either individually or in combination) or when transfected with empty vector (EV). Overexpression of syndecans was validated in Western blot (Supplemental Fig. 3b) and overexpression of HS by confocal microscopy using mAb 10E4 (Fig. 5a). Compared to corresponding control cells, syndecan-overexpressing cells showed increased virus uptake/transduction (Fig. 5b). In these terms, Synt-CRISPR MCF-7 cells over-expressing syndecan were no longer significantly different from control MCF-7 cells expressing syndecan at endogenous levels (Fig. 5b). Yet, even when displaying high levels of syndecan and HS, Synt-CRISPR MCF-7 cells were more difficult to transduce than SDC-overexpressing control MCF-7 cells, indicating still other ‘virulence’ factors remain limiting in syntenin-KO cells. Like syndecans, CD63 composes direct cargo for the PDZ domains of syntenin^41^. Also the levels of CD63 are lowered in syntenin KO cells (Fig. 2b, Supplemental Fig. 3a). Recently, CD63-syntenin complexes have been implicated in the post-endocytic trafficking of oncogenic papilloma viruses^42^. Of note, human papilloma virus L1-like vesicles bind to syndecans^43^. Double transfections of WT and Synt-CRISPR MCF-7 cells with LUC IRES eGFP virus and mCherry or mCherry-CD63 indicate that CD63 plays no role in retroviral transduction as tested here (Supplemental Fig. 5). Thus, factors other than syndecan and CD63, most likely syntenin itself, remain limiting for that process in these cells.

The latter contention was explored by testing whether syntenin (re)expression rescues retroviral transduction. WT MCF-7 cells and Synt-CRISPR MCF-7 cells were first transfected with plasmid encoding mCherry (empty vector), mCherry-syntenin WT (wild type syntenin), mCherry-syntenin ∆ALIX (syntenin with triple LYP to LAA mutations, defective in ALIX-binding and exosome formation^23^) or mCherry-syntenin K215A (syntenin defective in PIP2-binding and cargo recycling^44^) and, 2 days later, with LUC IRES eGFP virus. Two days after retrovirus infection, cells expressing mCherry were monitored for eGFP expression (Fig. 5c). As expected, when transfected with empty vector, significantly less Synt-CRISPR than WT MCF-7 cells were virally transduced (and expressing eGFP). Compared to empty vector, the syntenin with mutant LYP motifs had no significant effects on the eGFP expressions. In contrast, both the expressions of wild-type syntenin and recycling-defective syntenin significantly enhanced the eGFP-expression. These effects were most pronounced in Synt-CRISPR MCF-7 cells. Moreover, when over-expressing wild type or recycling-defective syntenin the eGFP expressions of WT MCF-7 cells and Synt-CRISPR MCF-7 cells were not significantly different. These results indicate that the functions of syntenin in linking membrane cargo to machinery involved in endosomal membrane budding and fission (via ALIX binding) play a major role in viral transduction, prevailing over additional syntenin involvements in receptor recycling and HS cell surface expression.

## Discussion

In this study, we identify a second, complementary role of syntenin in sustaining vesicle-mediated cellular exchanges. Syntenin not only supports the endosomal budding of cargo and exosome production, but also controls the uptake of exosomes and the effectiveness of viral transductions. Syntenin controls the expression levels of syndecans and CD63, both composing direct cargo for the PDZ domains of syntenin. Both syndecans and CD63 are involved in loading exosomes with cargo in exosome-producing cells. By also controlling exosome uptake and exosome-like virus-mediated exchanges in recipient cells, possibly including post-uptake events, syndecans and CD63 are both also involved in retrieving cargo from exosomes. Yet, surprisingly, more than affecting the abundance of cell surface receptors involved in vesicle docking and fusion, it is the role of syntenin in linking membrane cargo to the ESCRT machinery, via ALIX, that prevails in viral transduction. Syntenin thus appears to occupy a central place in exosomal pathways.

Despite the evidence for the above, but consistent with previous studies in mice by others^45,46^, syntenin-KO mice are viable, show no major defects and have normal fertility. This is surprising, given the broad expression of the syntenin protein in fetal and adult human^27^ and mouse tissues^47^ and the lethality of the corresponding morpholino-mediated knock downs in zebra fish and *Xenopus*, where syntenin is required for polarized early embryonic cell movements^48,49^. Moreover, in zebrafish, the yolk syncytial layer releases extracellular vesicles with exosome features into the blood circulation, in a syntenin-dependent manner. These exosomes are captured and endocytosed by patrolling macrophages and endothelial cells, and interference with the biogenesis of these exosomes suggests a role in trophic support of the caudal venus plexus of the animals, demonstrating functional inter-organ communication by exosomes^50,51^.

Yet, as shown here, syntenin deficient mice may be ‘refractory’ to (some forms of recombinant) viral transduction. By extension, we surmise these animals might also be ‘resistant’ to some forms of exosomal communication. Clearly, both contentions remain to be formally proven in further experiments. Yet, our preliminary observations in these mice might be consistent with the evidence that AAV can associate with exosomes, and that exosome-associated AAV vector is more efficient at gene delivery in the brain, at low vector doses, relative to conventional AAV^52,53^. As reported by Baietti et al. for the siRNA-mediated knock down of syntenin in MCF-7 cells^23^, and here also for MCF-7 cells with a stable CRISPR/Cas9-mediated syntenin gene inactivation, MEFs derived from syntenin-KO animals show reduced release of exosomal markers like CD63, ALIX and syndecan-1 C-terminal fragment. The results also confirm that other exosomal marker proteins such as CD81 remain unaffected, as reported by Roucourt et al.^34^. Thus, these results indicate that syntenin is an important and essential component of an exosomal pathway that is supporting the formation of a specific class of vesicles, or the loading of exosomes with specific cargo, or both. Thus, by extension, our results imply that precisely the syntenin pathway plays an important role in vesicle-mediated communication/viral transduction.

Yet, the problem of syntenin-negative cells extends beyond the simple lack of exosome production. Our results also reveal lower viral transduction efficiency and a decrease in exosome uptake in syntenin-KO MEFs and in syntenin-negative, CRISPR-engineered MCF-7 cells. This is consistent with the suggestion that retroviruses and exosomes might share similar mechanisms of dissemination^54–56^, including similar entry pathways in host cells^57^. Numerous studies show that several viruses, including HIV-1 (an exosome-like retrovirus), exploit heparan sulfate proteoglycans for uptake in cells^58,59^. Various macromolecular complexes, including viral particles and lipoproteins, use HSPGs to gain entry into cells^38,40^. Syntenin-KO MEFs and syntenin negative (CRISPR-engineered) MCF-7 cells show reduced HSPG expression. These findings are consistent with the known functions of syntenin in recycling syndecans to the plasma membrane^36^, but our data suggest that syntenin may also affect syndecans at RNA level. The mRNA levels of all four syndecans are significantly reduced in primary cultures of MEFs with syntenin-KO. Possibly syntenin-KO selects for the outgrowth of different types of MEFs or for MEFs with different states of activity, showing differential syndecan expressions. Reduced expression could also be due to RNA instability/degradation or to reduced transcription, by indirect or more direct mechanisms; e.g. syntenin binding the transcription factor Sox4 in the nucleus and modulating transcriptional output^60^. So far, the mechanism by which syndecan mRNA levels are affected in syntenin-KO MEFs is unexplained, and needs to be further addressed. Clearly, sustaining syndecan expressions, syntenin appears to be instrumental in maintaining the expression levels of some of its most important peptide interaction partners, in what could be considered as a feed-forward regulatory mechanism.

Attempts at rescuing syndecan expressions in syntenin-negative cells enhanced retroviral transduction. This suggests that the presence of HSPGs on the cell surface is important for mediating uptake and entry of retrovirus particles, although it is possibly (if not likely) not the sole factor involved. Indeed, syntenin-deficient cells with ‘normalized’ syndecan expressions are still relatively resistant to retroviral transduction. Moreover, recycling-deficient syntenin suffices for rescuing virus susceptibility. Further studies should more specifically address whether viruses/exosomes are less able to adhere, enter or fuse with syntenin-negative cells. Yet, two considerations can already be made in this context. First, it is interesting to note that syntenin also binds avidly to nectin-1^61^, which, next to 3-O-sulfated HS^38,40^ (possibly provided by syndecan^62^) is one of the entry receptors for HSV. Second, enveloped viruses like vesicular stomatitis virus infect cells through endosomes. There, the viral envelope undergoes fusion with endosomal membranes, thereby releasing the nucleocapsid into the cytoplasm and allowing infection to proceed. Of note, at least for vesicular stomatitis virus, the viral envelope fuses preferentially with the membrane of vesicles present within multi-vesicular endosomes. Then, these intra-endosomal vesicles (containing nucleocapsids) are transported to late endosomes, where back-fusion with the endosome limiting membrane delivers the nucleocapsid into the cytoplasm^63^. Conceivably, the fusions of endogenous ILV and endocytosed vesicles, with each other or limiting membranes or both, might be relatively rare stochastic events that depend on vesicular crowding (concentration) in endosomes. Thus, in such a scheme the lack of (syntenin-dependent) ILVs in endosomes might impede the fusion of viral envelopes with endosomal membranes. Alternatively, syntenin-ALIX might not solely be involved in membrane budding and vesicle abscission, but also actively participate in vesicle back-fusion, in effect the reverse of the process of ILV formation. Although unexplained, such a role has already been proposed for ALIX before, possibly involving functions at the limiting membrane of the multi-vesicular endosome, i.e. the creation of a hot-spot for ILV docking on the luminal side of that membrane, and ALIX interactions with lipids, such as LBPA^64^ and proteins that remain to be identified but likely include proteins participating in the formation of ESCRT complexes^65^. By inference, potentially these proteins include syntenin and syntenin docked to particular membrane cargo. Note that syntenin-ALIX docking to syndecan-CTFs (and not intact syndecans) would respond to the postulated need, for ILV back fusion to occur, to clear the intraluminal face of the limiting membrane bilayer of its glycocalyx-like cover^66^.

Interestingly, another group has inversely proposed that knockdown of syntenin expression increases HIV-1 cell fusion and viral entry^67^. It may be important to note that these contradictory results were obtained in CD4^+^ T cells. CD4^+^ T cells constitute the major HIV target cell type, but express no syndecans, using alternative, HSPG co-receptor-independent entry mechanisms^68^. The contradiction suggests there might be more than one pathway for HIV uptake and entry or aspect to this issue.

In conclusion, our current data suggest that syntenin not only takes part in the biogenesis of exosomes, but also participates in the uptake of exosomes and the exchange of viral/exosomal cargo between cells, indirectly, by impacting on syndecans and potentially also other uptake/entry receptors, but also directly, by its effects on the organization and dynamics of endosomal membrane domains. Also, its function on the ‘recipient side’ may result in stimulated signaling or transfer of pathogenic molecules. In such context, syntenin may thus be at the heart of a vicious circle that is sustained by exosomes. All this seems to justify further in-depth investigations on the role of syntenin in exosome biogenesis and uptake (including possible effects on the intercellular spreading of tau) potentially underpinning the significance of syntenin as a drug-target.

## Materials and methods

### Stereotaxic injection and immunohistochemical analysis of tau expression

AAV6-tau4R-P301L virus expresses human tau4R isoform carrying a P301L mutation under the control of the synapsin-1 promotor^15^. The left hemisphere of anesthetized mice (ketamine/xylazine, i.p. 0.1/0.05 g/kg body weight) was injected with AAV6-tau4R-P301L viral particles, stereotactically, at coordinates posterior 2.0 mm, lateral 1.0 mm, ventral 2.25 mm relative to bregma. A total of 10E8 transducing units of AAV6-tau4R-P301L virus in 2 µl were injected, at a rate of 0.2 µl/min, using 10 µl glass syringes with a fixed needle (Hamilton, Reno, Nevada). After injection, the needle was left in place for 5 min before withdrawal. Human tau expression was analyzed 2 months after injection, as described before^69,70^. Briefly, mice were anesthetized using pentobarbital (Nembutal) and perfused transcardially using ice-cold saline solution for 2 min. Brains were removed rapidly and fixed overnight in 4% paraformaldehyde. Fixed brains were stored in PBS + 0.1% sodium azide for subsequent immunohistochemical analysis on 40-µm free-floating coronal vibratome sections. Human tau expression was analyzed by immunohistochemistry, using biotinylated mouse monoclonal antibody HT7 (Thermo Scientific, MN1000B). Immune reactions were developed using streptavidin–horseradish peroxidase complex (Labconsult, Vectastain Elite ABC kit standard PK-6100) for detection of the biotinylated antibody and diaminobenzidine (MP Biomedicals, CAT#0898068) as chromogen. Images for the analysis of human tau expression were collected with Leica DMR microscope (objective HCX PL Fluotar 1.6 ×/0.05) using a high resolution colored CCD camera CD500 (exposure time 20 ms; software IM500). Expression of human tau was quantified using Image J software from the National Institute of Health (NIH) on sections around the injection site, measuring the number of pixels with a grey value above an arbitrary set threshold (taken as positive signal for tau expression). These experiments were approved by the Ethical Committee for Animal Experimentation of the KU Leuven, Belgium. All applied methods in these animal experiments were performed in accordance with the relevant guidelines and regulations.

### Expression vectors and reagents

Plasmids encoding mCherry or mCherry C-terminally fused to wild-type (WT) or mutant (∆ALIX and K215A) mouse syntenins, and mCherry-CD63 were as reported before^23,34,44^. For syntenin gain-of-function in MEFs, a cDNA encoding full-length non-tagged wild-type mouse syntenin and enhanced green fluorescent protein (Synt IRES eGFP) was cloned in bicistronic pMSCV viral vectors. Luciferase and eGFP (LUC IRES eGFP) were used as control. Open reading frames were confirmed by sequencing. Mouse monoclonal antibodies against the intracellular domain (ICD) of syndecan 1/3 (2E9) and syndecan 2 (6G12), and against remnant desaturated (delta) HS after heparitinase (lyase) digestion (3G10) and native HS (10E4), and rabbit polyclonal antibodies against ALIX (Rb67) and mouse syntenin (Rb96) were all described before^23,71–73^. Other antibodies were from commercial sources; antibodies against mouse CD63 (R5G2) was from MBL; antibodies against syndecan 4 ICD (Abnova, PAB9045), α-tubulin was from (Sigma-Aldrich), against β-actin (AC-15), EGFR (Cell signaling 2232), β1 integrin (BD Pharmingen 553715), fibronectin (BD Transduction 610077 Clone 10), CD81 (D4), anti TSG 101 (C20) and anti-HSP70 (W27) and anti GFP were purchased from Santa Cruz.

### Western blotting

Cells were plated in 10 cm diameter dishes. After 48 h, cell lysates were fractionated by SDS-PAGE and transferred to nitrocellulose membranes (GE Healthcare Life sciences). The membranes were blocked with 5% fat free milk and incubated with primary antibodies and then with HRP-conjugated secondary antibodies. The membranes were washed with PBS/0.1% Tween 20 buffer and antibody binding was revealed using enhanced chemoluminescence (ECL) reagent (Thermo Scientific) according to the recommendations of the manufacturer. Signals were detected on photographic films (GE healthcare).

### HSPG digestion

For imaging experiments, cells were cultured in Lab-Tek™-glass chambers (Thermo scientific), fixed in 0.4% PFA and washed in PBS before treatment with heparitinase and chondroitinase ABC. For Western blotting, cell lysates were prepared and 100 µg of total cellular protein was treated with or without 0.1 U/mL of heparitinase and 0.004 U/mL of chondroitinase ABC in digestion buffer (100 mM NaCl, 50 mM Hepes pH7, 1 mM CaCl_2_, 0.1% Triton X100, BSA 50 µg/ml with protease inhibitor cocktail, prepared freshly) for 3 h at 37 °C as in^23^. Reaction was stopped by washing the cells or by adding sample buffer and protein loading dye.

### Immunofluorescence staining and confocal microscopy

Cells were cultured on glass coverslips, fixed with 4% PFA for 15 min, washed in PBS and then incubated with 10E4 (1:200) or 3G10 (1:200) antibody in PBS containing 0.3% BSA and 0.05% saponin. Coverslips were mounted in DABCO/Mowiol and observed with a Zeiss Meta confocal microscope (LSM 510 META, Zeiss and Olympus FluoView FV1000) with a UV laser and a 60 × objective. Confocal images were analyzed and mounted using Photoshop (Adobe, San Jose, CA, USA) software.

### Exosomes and total cell lysates

For comparative analyses, exosomes were collected from equivalent amounts of culture medium, conditioned by equivalent amounts of MEF cells, for equivalent lengths of time. After 24 h, the cells were washed three times with PBS and refreshed with MEF media containing 10% exosome-depleted FCS. Cell-conditioned media were harvested 16 h later. Exosomes were isolated from these media by differential centrifugation in three steps at 4 °C: 10 min at 500*g*, to remove cells; 30 min at 10,000*g* to remove cell debris; and 3 h at 100,000*g*, to pellet exosomes, followed by one wash (suspension in PBS/centrifugation 1 h at 100,000*g*), to remove soluble serum and secreted proteins. Exosomal pellets prepared by differential high-speed centrifugation were then re-suspended in lysis buffer (PBS supplemented with 1% NP40, 10 mM EDTA and protease inhibitor cocktail (Roche). The corresponding cells were washed in cold PBS and scraped on ice. Lysates from corresponding cultures were cleared by centrifugation at 300×*g* for 5 min at 4 °C and then re-suspended in lysis buffer. Equal volumes of total lysates and exosomal proteins were fractionated by SDS-PAGE and analyzed for exosomal marker proteins by Western blotting. For nanoparticle analysis, aliquots of the exosomal preparations were resuspended in PBS and analyzed at similar dilutions in a Nanosight NS-300 instrument (Malvern). For each sample, three videos of 60sec were recorded at 25 °C and at a concentration of 20–60 particles per frame and used to calculate mean values of particle concentration and size.

### Exosome uptake

Exosomes loaded with eGFP-tagged syntenin (eGFP-labeled exosomes) were isolated from stably transfected MCF-7 cells with doxycycline-inducible eGFP-syntenin expression, as reported before^34^. Aliquots of 100 µl of exosome-free MEF medium were supplemented with eGFP-labeled exosomes (50 µg of total protein) and were added to each well in labtek glass chambers (Thermo scientific). Cells were incubated for 8 h, washed (1 × PBS), fixed (4% PFA), permeabilized and stained with DAPI (nuclei) and 10E4 (HS). Uptake of eGFP-syntenin was analyzed by confocal fluorescence microscopy.

### Viral infections and FACS analysis

LUC IRES eGFP construct in pMSCV vector was used to transfect ecotropic and amphotropic phoenix packaging cells, for mouse and human transduction respectively. Viral supernatants were harvested after 24 h interval and used to transduce MEF and MCF-7 cells for 48 h. Cells were trypsinized and analyzed in flow cytometry experiments. Signals were quantified using FACS by gating eGFP positive cells that expressed the eGFP after viral transduction with LUC IRES eGFP construct. Values are expressed as percentages of transduced cells. The signals were corrected for the background signal caused by auto fluorescence. A total of 20,000 events were recorded for each condition, in three independent experiments. For HSPG rescue experiments, MCF-7 cells were transfected to overexpress full length syndecan cDNAs cloned in pcDNA3.1/Zeo + vector (all syndecans individually and also in co-transfection), and analyzed in flow cytometry experiments after viral transduction; empty vector was used as control. For CD63 rescue experiments MCF-7 cells were transfected to overexpress full length mCherryCD63, alone or in combination with a mixture of all four syndecans, and further analyzed in flow cytometry experiments after viral transduction.

### Statistical analysis

Statistical analysis was performed using the standard two-tailed Student’s *t* test, and *P values < 0.05 (*), < 0.01 (**) and < 0.001 (***) were considered statistically significant. Metamorph, Image J and ColonyDoc-It acquired data were processed with GraphPad Prism software.

## Supplementary Information

Supplementary Information.
