# Supplementary material for: Syntenin-knock out reduces exosome turnover and viral transduction
Source: Sci Rep. 2021 Feb 18;11:4083. doi: 10.1038/s41598-021-81697-4 (PMC7892569; doi:10.1038/s41598-021-81697-4)

Syntenin-knock out reduces exosome turnover and viral transduction.

Rudra Kashyap[1,2], Marielle Balzano[2], Benoit Lechat[1], Kathleen Lambaerts[1], Antonio Luis Egea-Jimenez[2], Frédérique Lembo[2], Joanna Fares[2], Sofie Meeussen[1], Sebastian Kügler[3], Anton Roebroek[1], Guido David[1] and Pascale Zimmermann*[1,2].

1 Department of Human Genetics, KU Leuven, Leuven, Belgium.

2 Centre de Recherche en Cancérologie de Marseille (CRCM), Equipe labellisée LIGUE 2018, Aix-Marseille Université, Marseille, F-13284, France, Inserm, U1068, Institut Paoli-Calmettes, CNRS, UMR7258, Marseille, France.

3 Department of Neurology, University Medicine Göttingen, Göttingen, Germany

*Correspondence should be addressed to Pascale Zimmermann.

Centre de Recherche en Cancérologie de Marseille

27 bd Leï Roure BP 30059

13273 MARSEILLE CEDEX 09

Phone: + (33) 4 86 97 73 51

Fax + (33) 491 26 03 64

e-mail: pascale.zimmermann@kuleuven.be or pascale.zimmermann@inserm.fr

Supplementary data.

Supplementary materials and methods

Construction of the syntenin targeting vector and generation of syntenin KO mice

Starting from a cosmid library, two consecutive BglII genomic fragments, of about 2.5 and 3.6 kbp, respectively, were used to generate a floxed syntenin targeting vector for the conditional inactivation of the syntenin gene. The 5' BglII fragment encoding the 3'end part of intron 1 (about 2.2 kbp), syntenin exon 2 (66 bp, containing the start ATG) and the 5'end part of intron 2 (about 250 bp) was used to introduce a *Lox*P site (5'ATAACTTCGTATAGCATACATTATACGAAGTTAT3') 166 nucleotides upstream of exon 2. By site directed mutagenesis, two unique restriction sites, NheI and SalI, were generated using the paired oligonucleotides (21-49, 21-50), to allow insertion of the *Lox*P sequence. The final targeting construct was assembled in a pBluescript vector. This was done by insertion of a 2.2 kbp BamHI fragment encoding the second *Lox*P site and a hygromycin cassette flanked by FRT sites downstream of this 5'-end fragment. This encompassing the about 2.0 kbp left vector arm. Then, the right vector arm was constructed by inserting the 3.6 kbp BglII fragment, encoding the 3'end part of intron 2, exon 3 and intron 4. The 8.3 kbp targeting insert of the targeting vector was isolated from the vector with NotI and KpnI, purified and concentrated by ethanol precipitation to a concentration of 1µg/µl. From here on, we made use of the services provided by InGenious Targeting Laboratory, Inc (ITL). The targeting construct was introduced into 129 SvEv embryonic stem (ES) cells by electroporation. Resistant cell clones were selected; DNA was prepared and subjected to PCR analyses. The region of interest was amplified using the primer set [5'GTCCTGAAACTTGCTGTG3' (sense, localized outside the targeting construct, immediately upstream of BglII in intron1) and 5'GGATAAAGAGACATTTCTGC3' (antisense, in exon 2), followed by two (semi-)nested PCR's [primer pair 5'CCAAGATGAATGTAGTTGGG3' (sense in intron 1) and 5'GGATAAAGAGACATTTCTGC3', and primer pair

5'CTGTTGGCACTGTTGGTAGAAG3' (sense in intron 1) and 5'GTATGCTATACGAAGTTATGC3' (antisense in *Lox*P)] to confirm amplification of the expected syntenin gene region and presence of the 5' *Lox*P site, indicative for homologous recombination. Candidate clones were confirmed by analysis with the primers (5'GTCCTGAAACTTGCTGTG3' and 5'GTATGCTATACGAAGTTATGC3'). Next, high molecular weight DNA of candidate clones with a homologous recombination was analyzed by Southern blotting to confirm the proper occurrence of the desired homologous recombination events. Eventually, two independent ES cell lines were injected into C57BL/6 blastocysts and transferred into pseudo-pregnant females to generate chimeric offspring. Mice were backcrossed for 9 generations and inter-crossed with a Cre deleter strain to obtain a syntenin KO allele. Next to a first primer pair (5'CCAAGATGAATGTAGTTGGG3' and 5'GGATAAAGAGACATTTCTGC3'), an additional primer pair (5'GACAGTTTGGTAACAGCTAGC3' and 5'CTCTCGATCGAGAATTCCG3') was used to check for correct deletion of sequences encoding exon 2 between the *Lox*P sites. The animal experiments to generate syntenin KO mice and their subsequent phenotypic characterization were approved by the Ethical Committee for Animal Experimentation of the KU Leuven, Belgium. All applied methods in these animal experiments were performed in accordance with the relevant guidelines and regulations.

Southern blot analysis of ES-cell clones

Both the 5' and 3' probes used in Southern blots were located outside the flanking regions present in the targeting vector and consisted, respectively, of a 392 bp and a 287 bp PCR fragment generated using the primers (5'CCTAAGGTCCAGATGAGA3', 5'GTTCTATGTCTTGGGTGG3' and 5'ATGGTCTCTGGAGCAC3', 5'CTCAGTGCAGTTATACAG3'). PCR fragments were purified with the QIAquick gel extraction kit (Qiagen) and radio-labeled with α^{32}P-dCTP using the Megaprime^{TM} DNA

labeling System (Amersham Biosciences). Unincorporated nucleotides were removed by NICK™ Columns (GE Healthcare) according the recommendations of the manufacturer. To exclude additional integrations in random places of the genome we additionally used a probe for the hygromycin B cassette. Genomic DNAs of different ES-cell clones (15 µg) were digested overnight with the enzymes EcoRI, HindIII and XbaI, separated on a 0,8% agarose gel (TBE), depurinated (0,25 M HCl) and transferred overnight by capillarity to a Hybond N+ membrane (Amersham Biosciences) with 0.6 M NaOH. Membranes were washed in 2X saline-sodium citrate buffer (SSC) solution to remove any residual agarose and fixed in 0.1X SSC, 0.5% SDS for 30' at 65°C. After pre-hybridization for 5 hours in hybridization buffer (0.1 X SSC, 100x Denhardt solution, 10% SDS, 50% dextran sulfate, 1% heparin, 1% salmon sperm DNA), membranes were hybridized overnight at 60°C in hybridization buffer containing $\alpha^{32}P$ labeled probes at 4 X 10^6 disintegrations per minute (DPM)/ml. After treatment at 60°C in 0.1 X SSC, 0.5% SDS, radioactive signals on the blot were detected by a Storage Phosphor Screen (Molecular Dynamics)

PCR analysis of mouse genotypes, RT-PCR and qPCR

Tail biopsies were taken from three weeks old mice. The tail was solubilized overnight at 55°C in lysis buffer (0.1 M NaCl, 50 mM EDTA, 0.5% SDS, 50 mM Tris, 100 µg/ml of proteinase K). Proteins were precipitated by adding 5M NaCl to a 1.3 M final concentration, mixing and centrifugation for 15' at 14,000 rpm. After repeating this step, the DNA was precipitated by 100% ethanol, washed with 70% ethanol, dried and dissolved in TE buffer (10 mM Tris, pH 7.5, 1 mM EDTA).

For RT-PCR analyses, total mRNA was extracted from different tissues with the RNeasy mini kit, in accordance with the manufacturer's instruction (Qiagen). Reverse transcription was performed with 200 ng of total mRNA. The first strand of cDNA was generated with PrimeScript® RT reagent Kit (Takara Bio). Reaction mix was incubated for 15 min at 37°C,

followed by inactivation for 5 sec at 85°C. To detect syntenin transcripts, PCR was performed with Phire Hot Start DNA II polymerase (ThermoFisher) on 2 µl of cDNA solution with primer (MuSynt_RTPCR_For) and primer (MuSynt_RTPCR_Rev). Cycling conditions consisted of 30 cycles with denaturation steps at 98°C for 5 sec, hybridization steps at 57°C for 5 sec and an extension step at 72°C for 15 sec.

For quantification of the different syndecan transcripts in MEF samples by qPCR, reactions consisted of 10 µL of Power SYBR® Green PCR Master Mix (ThermoFisher), 6 µL of RNase/DNase-free water, 2 µL of 10 µM primer mix, 2 µL of cDNA diluted (1/10) in a total volume of 20 µL. Primer sequences are shown in supplementary table 1. Cycling conditions were as follows: 10 min at 95°C followed by 40 rounds of 95°C for 15 sec and 60°C for 60 sec. Melting curve analysis to determine the dissociation of PCR products was performed between 65°C and 95°C. Actin was used as a housekeeping gene for the normalization of syndecan gene expression. For data analysis, the raw threshold cycle (C_T) value was first normalized to the housekeeping gene for each sample to obtain ΔC_T. The normalized ΔC_T was then calibrated to control cell samples to obtain $\Delta\Delta C_T$. Experiments were performed in triplicates from which mean values were determined.

Mouse embryonic fibroblasts (MEFs)

MEF cells were isolated after sacrificing pregnant mice at 13.5 day post-coitum (d.p.c.), under aseptic conditions. Embryos were separated from the placentas and embryonic sacs. Heads and red organs were removed, and embryos were washed in PBS. Tissue was finely minced using a sterile razor blade until it became possible to pipette the suspension. Tissues were transferred into 10 cm Petri dishes containing 10 ml of pre-warmed freshly prepared MEF culture medium (components to make ~500 ml of medium: 450 ml of DMEM, 50 ml of FBS (10% (v/v)), 5 ml of 200 mM L-glutamine (1/100 (v/v)), 100 units/ml of penicillin, 100 µg/ml streptomycin, 0.1

mM beta-mercaptoethanol), and resuspended with the help of a 10 ml pipette. Medium was refreshed after 24 h. After 48 h, a major part of the passage zero (P0) cells were frozen for future usage. Remaining P0 cells were expanded in T175 flask(s) till P2 and stored further.

Generation of syntenin-negative MCF-7 cells by CRISPR/ Cas9 engineering

Cas9-targeted gene disruption was performed using methods similar to those reported elsewhere [1]. Briefly, oligonucleotides for guide RNAs (gRNAs) targeting syntenin genes were synthesized as 20 bp fragments, with the following sequences: Seq-CACCgCTATCCCTCACGATGGAAGT (gRNA-A) and AAACACTTCCATCGTGAGGGATAGc (gRNA-C), and were cloned into the oligo cloning site of pSpCas9 (BB)-2A-GFP (Addgene plasmid # 48138). The guide sequence containing Cas9 vector was transfected to MCF-7 cells. After 48 h of transfection, cells were selected based on GFP status using a cell sorter (Beckman Coulter) and colonies were allowed to form from single cells. Syntenin expression was analyzed by western blotting in 10 clones obtained from each gRNA-A and gRNA-C, of which 3 were found to have lost syntenin protein. In all cases, deletion was confirmed by PCR amplification, revealing deletions at the target site.

MCF-7 cells were obtained from ATCC. Cells were routinely grown in DMEM/F12 (1:1) medium (Life Technologies) supplemented with 10% fetal bovine serum (Gibco). Serum used for experiments was depleted of exosomes, by prior overnight centrifugation at 140,000xg. For transient expressions, the cells were transfected the day after plating using Fugene 6 reagent (Roche) as described before [2].

References

1 Imjeti, N. S. et al. Syntenin mediates SRC function in exosomal cell-to-cell communication. Proc Natl Acad Sci U S A 114, 12495-12500, doi:10.1073/pnas.1713433114 (2017).

2 Kashyap, R. et al. Syntenin controls migration, growth, proliferation, and cell cycle progression in cancer cells. Front Pharmacol 6, 241, doi:10.3389/fphar.2015.00241 (2015).

Legends to supplemental figures

Supplemental Figure 1 (related to Figure 1). Generation and validation of syntenin-knockout mice. a. Schematic representation of a relevant part of the murine syntenin gene, before and after homologous recombination (HR) with a targeting construct. Exon 2 contains the (ATG) start codon. The targeting construct used for generating animals with a modified syntenin allele allowing conditional inactivation of the gene (Synt-KO) contains exon 2 flanked by *Lox*P sites (black triangles), next to a hygromycin-resistance B cassette (Hyg B), flanked by FRT sites (open triangles), extended, both upstream and downstream, by regions of homology. Following electroporation and hygromycin B-selection, mouse embryonic stem cell clones with the desired targeted insertion (with both the LoxP sites and the hygromycin gene introduced into the syntenin locus by HR) were identified using PCR and Southern blotting and subsequently used for injection into blastocysts to generate floxed mice. Deletion of exon 2 *in vivo* was obtained by breeding the floxed mice with a Cre-expressing transgenic mouse line where expression of Cre was under the control of the Sox2 promoter. This resulted in the general deletion of exon-2 (i.e. in all tissues, from early development on). After Cre recombination, the inactivated locus still contained the FRT-flanked selection marker gene. S, B and Bg represent restriction enzyme sites for Sac I, Bam III and Bgl II respectively. **b.** PCR analysis of mouse tail samples to validate Synt KO mice. The PCR in the upper panel (primer set 66.08 and 66.07)

reveals the deletion of exon 2 sequences as indicated by lack of amplification for the KO allele, whereas the lower panel (primer set 81-97 and 81-98) shows the amplification of a small 88 bp fragment representing remaining sequences after recombination of the *Lox*P sites by Cre starting upstream of the 5' *Lox*P site and downstream of the 3' *Lox*P site (no amplification for WT allele). **c.** Syntenin mRNA expression, confirmed by two step RT-PCR in different tissues, from wild-type (WT) and Synt KO mice. cDNA from KO mice shows deletion of 51bp, corresponding to exon 2 in the syntenin transcripts. **d.** Western blot analysis of syntenin protein expression in primary cultures of fibroblasts derived from 13.5 day old wild-type (WT), heterozygous and Synt KO mouse embryos (MEFs). α-Tubulin was used as loading control. **e.** Quantification of litter size and viability by counting number of pups and number of viable pups respectively. Parameters were monitored in 9 KO/KO and in 8 WT/WT breeding pairs, for a total of 37 and 32 litters and 272 and 211 mice, respectively.

Supplemental Figure 2 (related to Figure 3). Loss of syntenin expression in primary MEFs leads to a decrease in SDC expression. a. (Left) Western blot analysis of heparitinase and chondroitinase ABC digested lysates from the primary MEFs stained for syndecan core proteins by mAb 2E9 (specific for the syndecan 1 (SDC1) and syndecan 3 (SDC3) intracellular domain (ICD)), mAb 6G12 (specific for the syndecan 2 (SDC2) ICD)) and rabbit anti-SDC4 (specific for the syndecan 4 (SDC4) ICD)). The arrowheads indicate the band with the appropriate molecular mass for each syndecan family member. The low molecular mass band indicated by the 'CTF' arrowhead likely represents the C-terminal fragment(s) of shed / proteolytically-processed endosomal forms of syndecan-1/3. (Right) Bar graphs represent the quantification (means) of three independent experiments, taking band intensities in WT cells as 100%. **b**. Bar graphs representing the qRT-PCR data on the relative *syndecan* (*SDC*) gene expressions in WT and Synt KO MEFs (taking the levels in WT cells as 100%).

Supplemental Figure 3 (related to Figure 4). Characterization of MCF-7 cells with syntenin-knockout. MCF-7 cells that lack syntenin were created by CRISPR/Cas9 technology (Synt-CRISPR MCF-7 cells). (**a**) Western blot analyses of the lysates (left) and exosomes (right) of control and Synt-CRISPR MCF-7 cells using antibodies directed against proteins as indicated. (**b**) Control and Synt-CRISPR MCF-7 cells were tranfected with empty vector or vector encoding the SDCs 1-4 (individually or in combination). Cell lysates were analyzed by western blotting, using the 3G10 mAb revealing residual delta HS after digestion of the HSPGs of the cells with heparitinase and chondroitinase ABC, present on core proteinstentatively identified as the SDC1-4.

Supplemental Figure 4 (related to Figure 4). Exosomes loaded with eGFP-syntenin. MCF-7 cells were stably transfected to express eGFP-syntenin under the control of doxycyclin. Western blot analysis of eGFP-syntenin protein expression in cell lysates and exosomes derived from control (non-induced) and doxycyclin-induced MCF-7 stable clones. Lysates and exosomes stained for eGFP (left) and syntenin (right; eGFP-Syntenin and endogenous syntenin). β-Actin was used as loading control.

Supplemental Figure 5 (related to Figure 5). CD63 expression does not boost retroviral transduction of wild-type or syntenin-negative MCF-7 cells. a. Retrovirus encoding LUC IRES eGFP, produced using phoenix packaging cells, was incubated for 48h with Ctrl MCF-7 cells and with Synt-CRISPR MCF-7 cells, over-expressing m-Cherry empty vector or mCherry-CD63. Fluorescent protein expressions were quantified by flow cytometry. eGFP expression in mCherry-expressing cells was taken as a measure of retroviral transduction. eGFP expression in mCherry-expressing control wt and Synt-CRISPR MCF-7 cells was taken as 100. $n = 3$, bars represent mean values ± SD; n.s., non-significant (Student's *t*-test). **b.** Retrovirus encoding LUC IRES eGFP was incubated for 48h with Ctrl MCF-7 cells and with Synt-CRISPR MCF-7 cells, transfected with empty vector or over-expressing syndecans and/or mCherry-CD63, in

the indicated combinations. Fluorescent protein expression was quantified by flow cytometry. The percentage of the cells expressing eGFP was taken as a measure of retroviral transduction. eGFP expression in wild type (control) MCF-7 cells transfected with empty vector was taken as 100%. Of note, co-transfection of CD63 with syndecan did not affect (enhance) viral transduction (percentage of eGFP expressing cells) in syndecan expressing cells (n = 1).

Supplemental figure 1

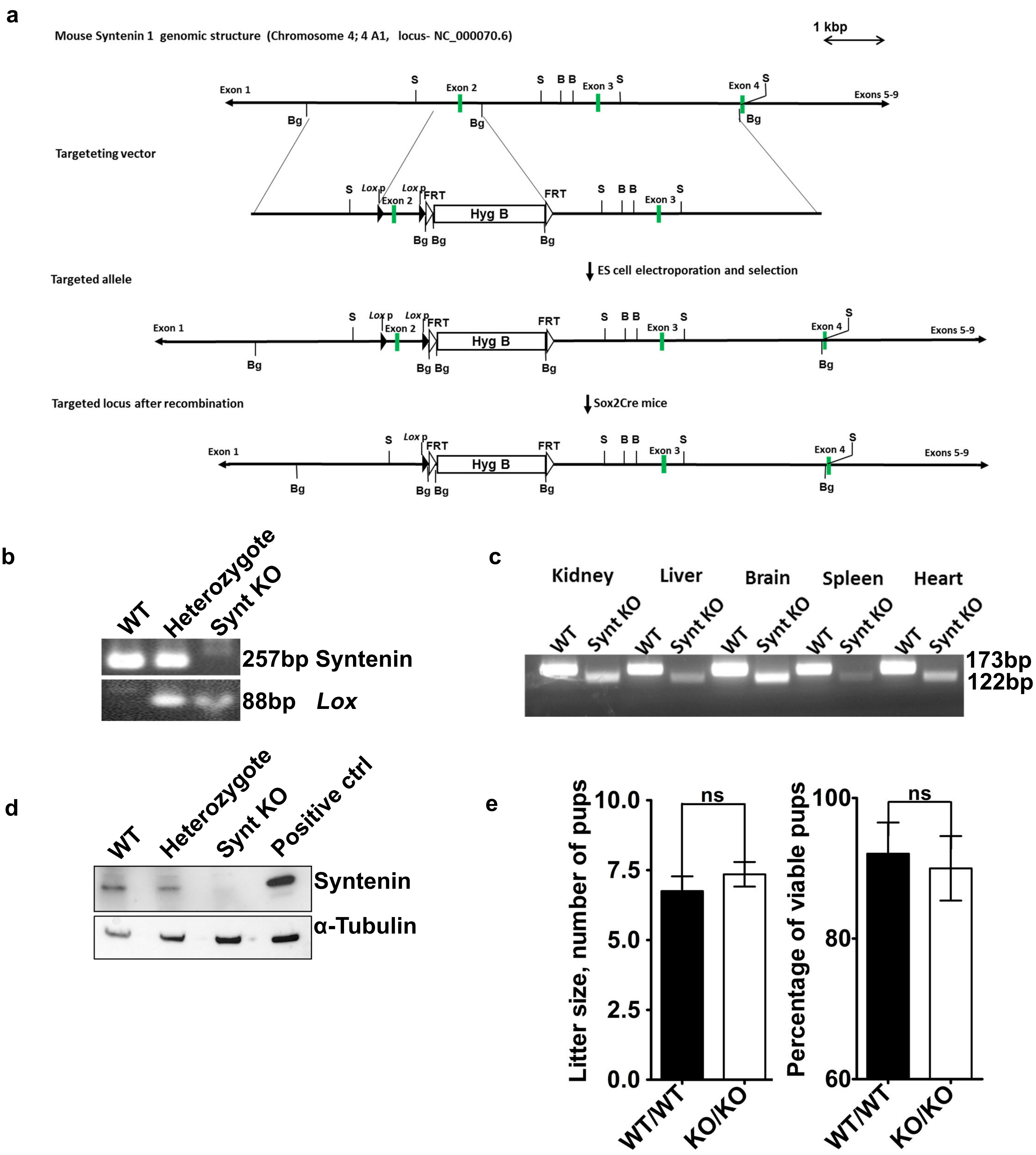

Supplemental figure 2

a

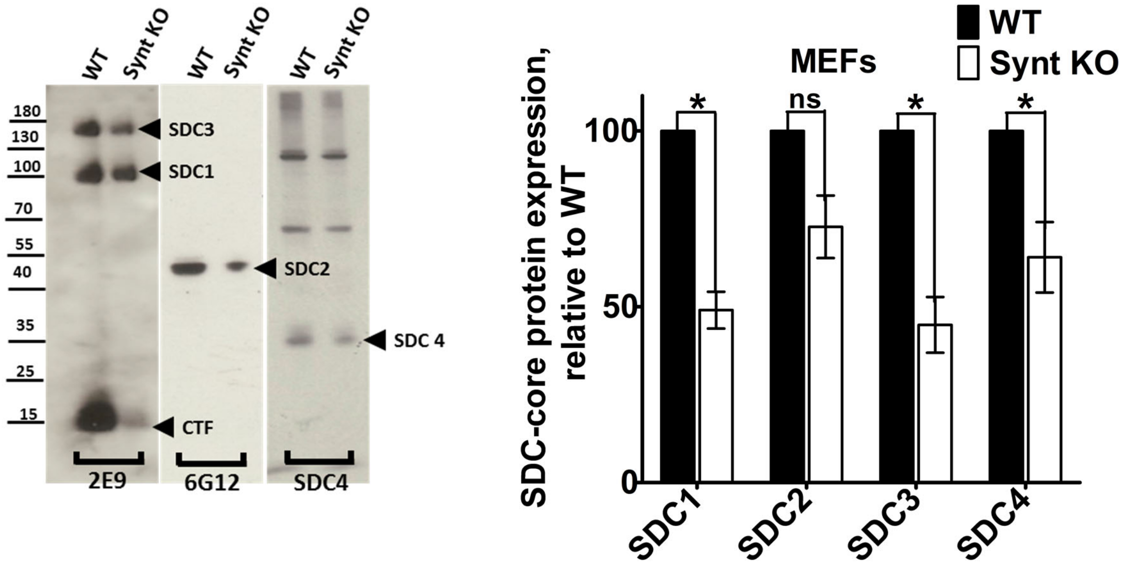

b

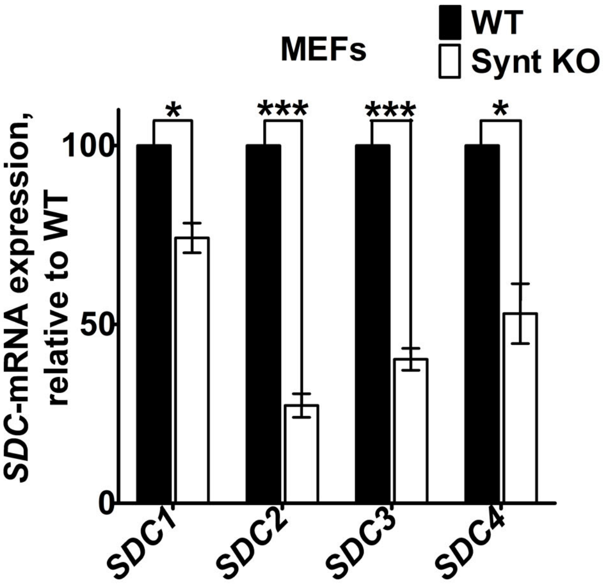

Supplemental figure 3

a

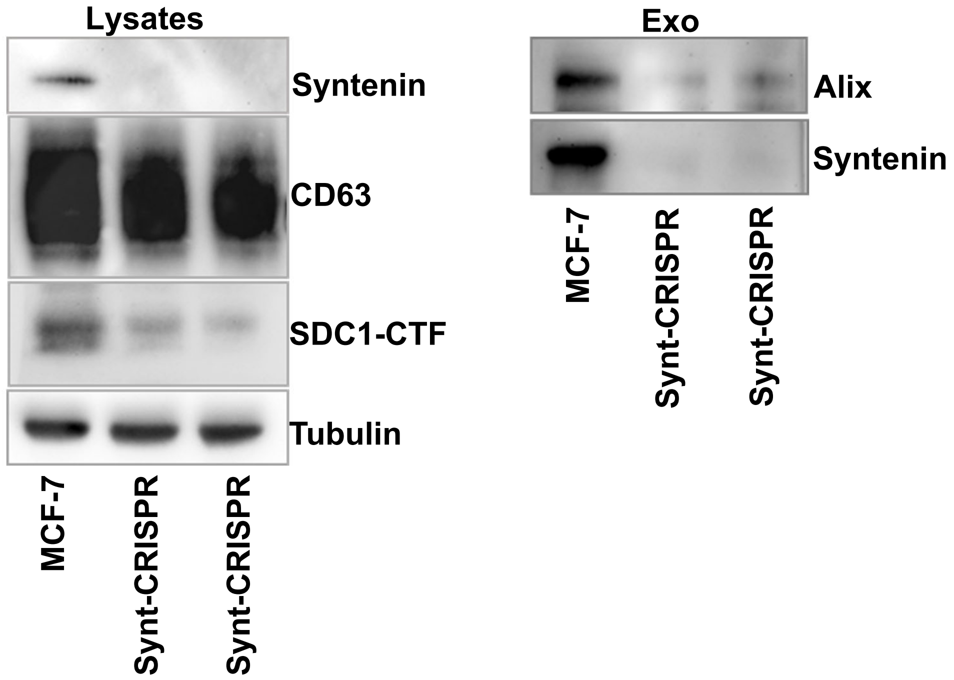

b

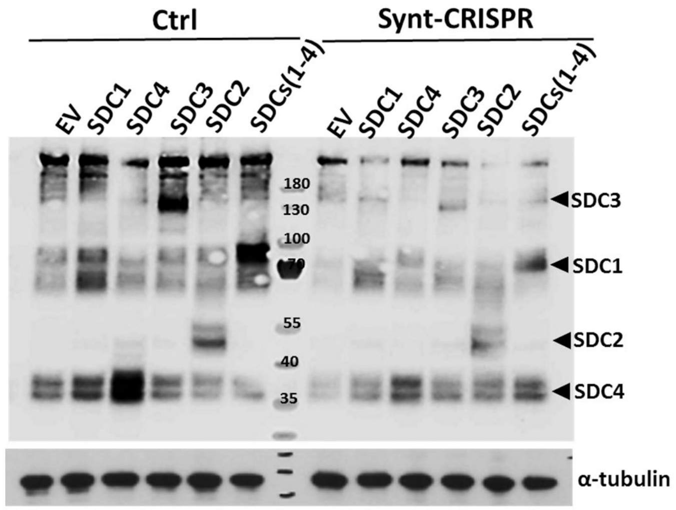

Supplemental figure 4

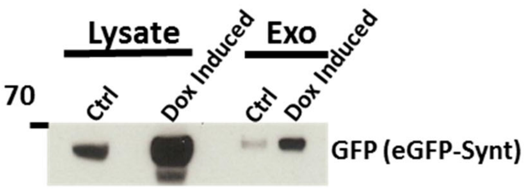

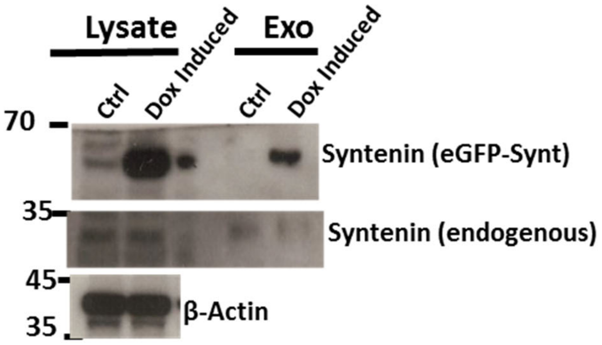

Supplemental figure 5

a

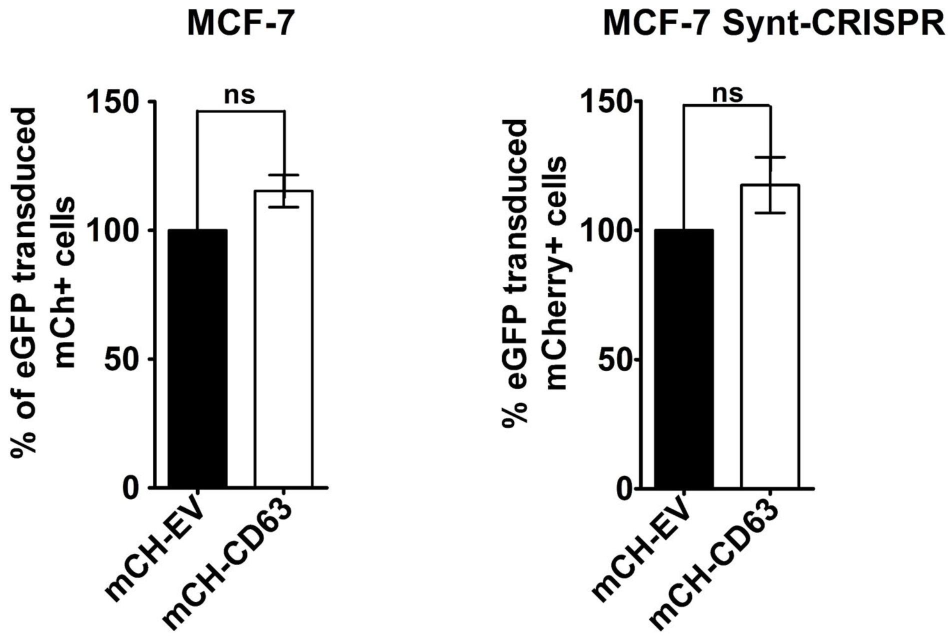

b

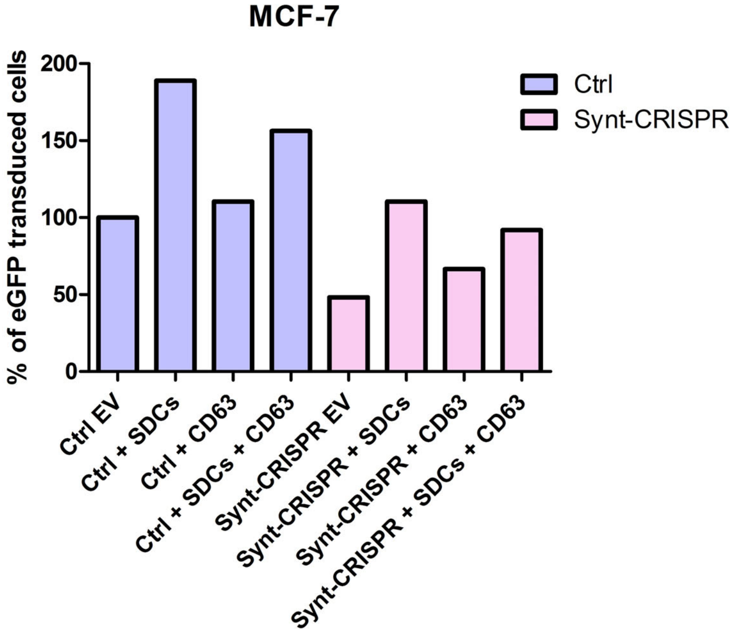

Supplement: Supplementary file 1 — Supplementary Information. [file 41598_2021_81697_MOESM1_ESM.pdf]
